# Cardiac Arrest in an Extensive Pulmonary Tuberculosis Patient

**DOI:** 10.7759/cureus.16057

**Published:** 2021-06-30

**Authors:** Vancy Zora

**Affiliations:** 1 Internal Medicine, Indiana University (IU) Health Ball Memorial Hospital, Muncie, USA

**Keywords:** mycobacterium tuberculosis, cardiac arrest, pulmonary tb, extrapulmonary tb, tb myocarditis, sudden cardiac death

## Abstract

Tuberculosis (TB) disease affects a large population worldwide but is often missed. Early diagnosis can be challenging at times due to failure to include TB in the differential diagnoses. Tuberculosis is a treatable disease but it can lead to undesirable consequences if left untreated. Here we report a case of a young Guatemalan woman presented with respiratory failure and extensive lung opacification on imaging who went into cardiac arrest. Post resuscitation, workup was significant for *Mycobacterium tuberculosis *(TB) on sputum smear. Despite appropriate treatment, she expired. The aim of this paper is to summarize TB complications that directly contribute to mortality as many clinicians do not consider TB as an immediate cause of death.

## Introduction

Mycobacterium tuberculosis (TB) infection is one of the most common infections globally. Nearly two billion people are infected with TB around the globe [1]. In fact, TB is the leading cause of death worldwide. According to CDC, approximately 10 million people develop TB each year and 1.6 million people die from it globally. In the United States (U.S.), TB infection often occurs among persons from Asia, Africa, Russia, Eastern Europe and Latin America [1]. Non-U.S.-born persons have had exposure to TB in their countries and latent TB is reactivated later in their lives. It is also worth noting that certain ethnicities are disproportionately affected by TB. According to CDC, approximately 87% of all TB cases in the U.S. occurred among persons who were Asian, Black or African American, Hispanic or Latino, American Indian or Alaska Native, Native Hawaiian or Other Pacific Islander [1]. It is thought that this disproportion is likely due to a greater proportion of people in these groups who also have other risk factors for TB [1]. TB risk factors include born in a TB-endemic country, HIV infection, low income and education status, and exposure to TB in certain settings such as homeless shelters, incarceration, and health care facilities.

## Case presentation

**Figure 1:**
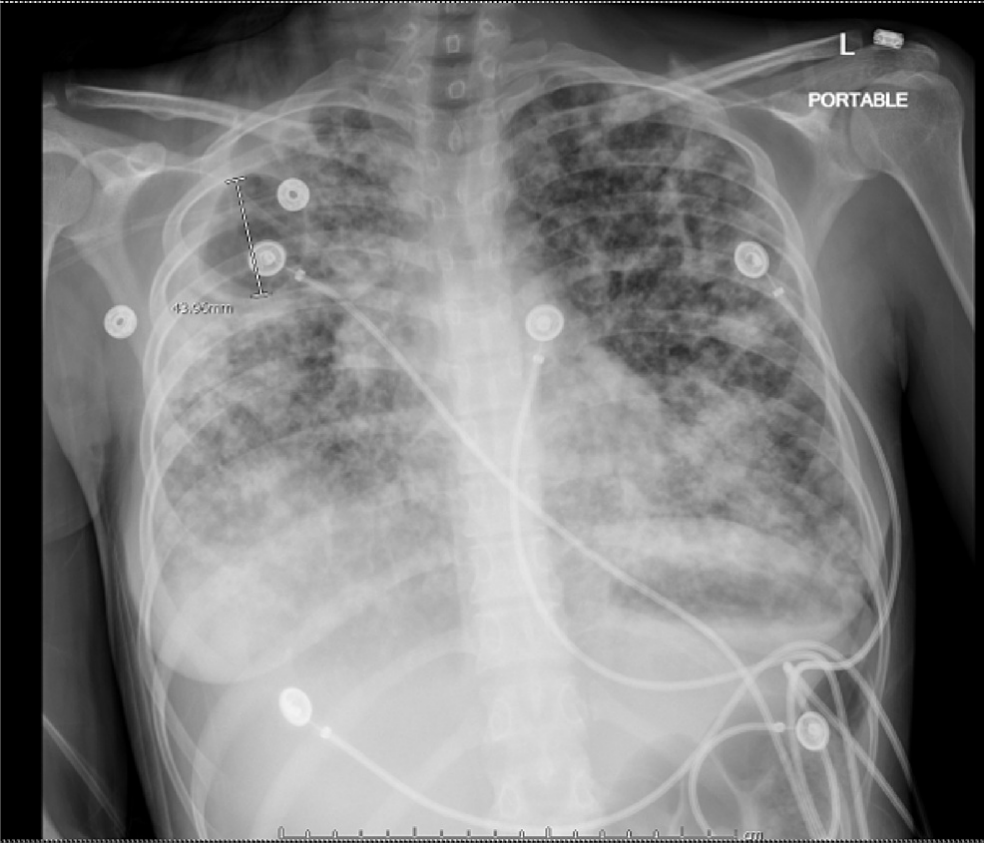
Chest radiograph revealing diffuse infiltrates with apical right cavitary lesion

**Figure 2:**
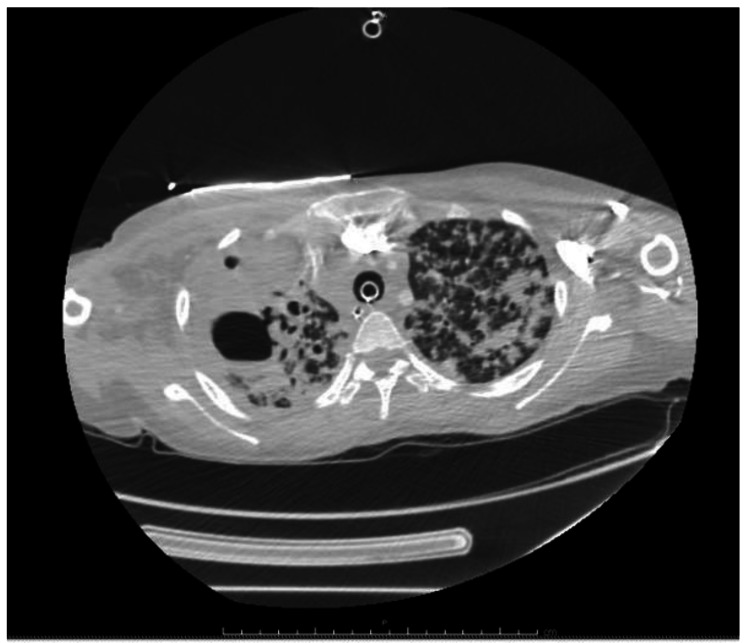
Computed tomography angiography (CTA): Extensive pulmonary opacities and right upper lobe cavitary lesion in a distribution most worrisome for post primary TB

**Figure 3:**
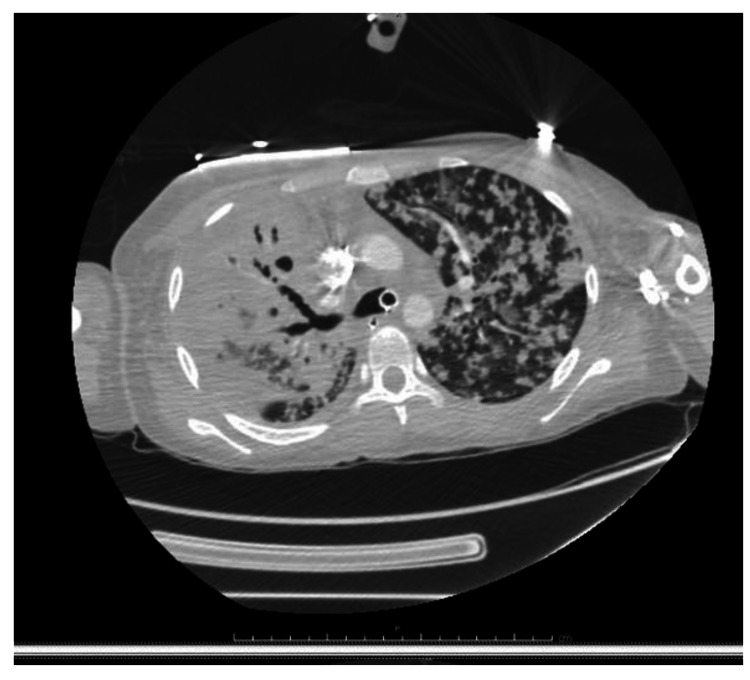
Computed tomography angiography (CTA): Extensive pulmonary opacities

A 21-year-old Guatemalan female presented to emergency department with dyspnea, fever, and cough for three days along with arthralgia and weakness for two months. She was three months post-partum. Her child experienced similar symptoms. She is gravida 2, para 2. Her oldest son born in 2016 in Guatemala and is in good health. On admission, she was afebrile, hypotensive, tachypneic and tachycardic. Chest X-ray revealed bilateral infiltrates (Figure 1). Non-invasive pulmonary ventilation was initiated. Two hours later, she became bradycardic which transitioned into asystole. Resuscitation efforts lasted 15 minutes in which she required one defibrillation for ventricular fibrillation until she was resuscitated. Computed tomography angiography (CTA) chest revealed 5.1 cm cavitary lesion in right upper lobe with complete opacification in the apex and extensive nodular opacities throughout the lungs (Figures 2, 3). The patient was admitted to intensive care unit in airborne isolation. Bronchoscopy showed yellow mucoid secretions in all airways. Sputum cultures revealed 4+ AFB in smear. Infection disease was consulted and she was started on Rifampin, Isoniazid, Pyrazinamide and Ethambutol 24 hours after admission. The patient remained unresponsive when sedation was turned off, therefore lumbar puncture (LP) was performed on day 4 of hospitalization. LP results showed clear and colorless fluid, 95% neutrophils/bands (high), 1% lymphocytes (low), 39 mg/dl glucose (low), 47 mg/dl proteins (high), 22/cumm total nucleated cell count (high). Head computed tomography (CT) without contrast was repeated and showed cerebral edema. Magnetic resonance imaging (MRI) without contrast showed severe anoxic brain injury but there was no discrete findings of central nervous system TB. Neurology was consulted and determined prognosis to be “nil”. Neurology advised to observe patient for the next few days and to obtain electroencephalogram (EEG). EEG showed extreme low aptitude consistent with severe diffuse cerebral dysfunction. On day 7, palliative care was consulted. After a long discussion with family members, family decided on comfort care extubation. Two hours after extubation, the patient comfortably expired.

## Discussion

TB complications are best understood in two categories, pulmonary and extrapulmonary. In most TB cases, patients die from pulmonary complications such as pneumonia, hemorrhage, airway compression, pulmonary edema, and pleural adhesions leading to respiratory failure and eventually death [2]. Extrapulmonary TB can also lead to death, particularly, if cardiac system is involved. For instance, if cardiac system collapses, so does the respiratory system and vice versa due to the synergetic relationship between the two systems. Other extrapulmonary TB involvement includes, nervous system (meningitis), abdominal (usually with ascites), skeletal (Pott's disease), scrofula (lymphadenitis), and genitourinary (renal) tuberculosis [3]. Here, we will mainly focus on cardiopulmonary complications.

In the pulmonary category, bronchopneumonia is the leading cause of death (64%) in TB patients followed by massive hemoptysis (30%) [4]. Any lesion, even if small and localized, has the potential for causing pulmonary hemorrhage. If pulmonary hemorrhage ensues, it can be fatal as even a small amount of blood can obstruct the airway. Additionally, pulmonary hemorrhage (if profuse bleeding) potentially leads to hypovolemic shock and asphyxiation from hemoaspiration. The causes of hemoptysis/pulmonary hemorrhage could be due to blood vessels rupture, a fistula between a blood vessel and an airway, mycetoma and in rare cases Rasmussen’s aneurysm - a branch of a bronchial or pulmonary artery that results in pseudo-aneurysmal dilatation due to inflammation [5].

TB involvement of the cardiovascular system accounts for 1-2% of TB deaths [6]. This is not limited to coronary vessels but includes the myocardium. TB complications of infected coronary vessels include premature MI due to granuloma formation [7] and progression of atherosclerosis leading to TB arteritis [8]. Conversely, TB myocarditis is a well-known cause of sudden cardiac death (SCD), predominately in young patients. Previously, it was thought that the myocardium involvement was due to systemic/miliary TB. However, majority of autopsy reports showed TB myocardia (TBM) without systemic involvement which led to other hypotheses. Some of the potential etiologies of TB myocarditis include, direct extension, lymphatic drainage, or direct spread from tuberculous pericarditis [8].

TB myocarditis is largely diagnosed after autopsy mainly because it is asymptomatic which makes pre-mortem diagnosis and intervention (such as implantable defibrillator) during life difficult. However, it should be suspected in any TB patient presenting with sudden cardiac arrest. Other cardiac presentations include, arrhythmias (atrial fibrillation, ventricular tachycardia and atrioventricular block), valvular dysfunction, congestive heart failure or superior vena cava obstruction [9]. In such scenarios, magnetic resonance imaging can be useful in detecting myocardium involvement. Anti-tuberculous medication therapy is the mainstay treatment for TB myocarditis. However, there is no evidence to suggest efficacy in SCD prevention.

In our case, it is unclear whether hypoxia led to cardiac arrest or if she had involvement of the myocardium at the time of presentation that triggered cardiac arrhythmia. However, the former rational is more plausible given her acuity of respiratory distress at the time of presentation. Yet, it remains uncertain if there was disseminated infection involving the cardiovascular system as autopsy was not performed. Additionally, it was unknown when the patient immigrated to U.S. or if she had TB testing at the time of her pregnancy.

## Conclusions

In the era of availability of effective therapy against TB, we continue to find a significant proportion of mortality among TB patients. TB can infect multiple organ systems best understood if divided into pulmonary and extrapulmonary infections. In the pulmonary category, bronchopneumonia is the leading cause of death. In the extrapulmonary category (mainly focused on cardiovascular system) infection can occur in the coronary vessels or the myocardium which is mostly diagnosed after death with autopsy. For a better control of TB infections and thus reduction in TB-related death, prevention and early diagnosis remain the mainstay. This encompasses setting standards of ethical and evidence-based policies for TB prevention, promoting and facilitating their implementation. Additionally, it is crucial to address the high rate of TB infection in non-U.S.-born population. In order to make a change, screening protocols of immigrants overseas must be improved, recent immigrants from endemic areas must be tested, close monitoring of TB-suspected immigrants, and ensure completion of treatment.

## References

[1] Centers for Disease Control and Prevention (2019). Epidemiology of Tuberculosis. https://www.cdc.gov/tb/education/ssmodules/pdfs/Module2.pdf.

[2] Chinen K, Ito K (2019). Sudden death caused by pulmonary fat embolism in a patient with miliary tuberculosis. Autops Case Rep.

[3] Haulman NJ, Hawn TR, Nolan CM (2008). Tuberculosis in travelers and immigrants. In: The Travel and Tropical Medicine Manual.

[4] Alkhuja S, Miller A (2001). Tuberculosis and sudden death: a case report and review. Heart Lung.

[5] Shih SY, Tsai IC, Chang YT, Tsan YT, Hu SY (2011). Fatal haemoptysis caused by a ruptured Rasmussen's aneurysm. Thorax.

[6] Leoni D, Rello J (2017). Cardiac arrest among patients with infections: causes, clinical practice and research implications. Clin Microbiol infect Dis.

[7] Rodríguez Y, de Armas Y, Capó V, Wissmann G, Goldani LZ, De Waard JH (2012). Sudden death related to tuberculous coronary arteritis. Int J Cardiol.

[8] Kinare SG, Bhatia BI (1971). Tuberculous coronary arteritis with aneurysm of the ventricular septum. Chest.

[9] Langara B, Georgieva S, Khan WA, Bhatia P, Abdelaziz M (2015). Case report: sudden cardiac death in a young man. Breathe.

